# Overview and factors associated with pregnancies and abortions occurring in sex workers in Benin

**DOI:** 10.1186/s12905-020-01091-6

**Published:** 2020-11-09

**Authors:** Gentiane Perrault Sullivan, Fernand Aimé Guédou, Georges Batona, Frédéric Kintin, Luc Béhanzin, Lisa Avery, Emmanuelle Bédard, Marie-Pierre Gagnon, Djimon Marcel Zannou, Adolphe Kpatchavi, Michel Alary

**Affiliations:** 1 Axe Santé des populations et pratiques optimales en santé, Centre de recherche du CHU de Québec, Université Laval, Québec, Canada; 2 Département de médecine sociale et préventive, Université Laval, Québec, Canada; 3 Dispensaire IST, Centre de santé communal de Cotonou 1, Cotonou, Bénin; 4 École Nationale de Formation des Techniciens Supérieurs en Santé Publique et en Surveillance Épidémiologique, Université de Parakou, Parakou, Bénin; 5 Medical Microbiology, College of Medicine, University of Manitoba, Winnipeg, Canada; 6 Département des sciences infirmières, Université du Québec à Rimouski, Lévis, Québec, Canada; 7 Département des sciences infirmières, Université Laval, Québec, Canada; 8 Faculté des sciences de la santé, Université d’Abomey-Calavi, Cotonou, Bénin; 9 Centre national hospitalier universitaire HMK de Cotonou, Cotonou, Bénin; 10 Département de Sociologie - Anthropologie, Faculté des Lettres, Arts et Sciences Humaines, Université d’Abomey-Calavi, Cotonou, Bénin

**Keywords:** Sex workers, Abortion, Pregnancy, Sub-Saharan Arica, Epidemiology

## Abstract

**Background:**

Behavioural and structural factors related to sex work, place female sex workers (FSWs) at high risk of maternal mortality and morbidity (MMM), with a large portion due to unintended pregnancies and abortions. In the African context where MMM is the highest in the world, understanding the frequency and determinants of pregnancy and abortion among FSWs is important in order to meet their sexual and reproductive health needs.

**Methods:**

Data from two Beninese cross-sectional surveys among FSWs aged 18+ (2013, *N* = 450; 2016, *N* = 504) were merged. We first performed exploratory univariate analyses to identify factors associated with pregnancy and abortion (*p* < 0.20) using Generalized Estimating Equations with Poisson regression and robust variance. Multivariate analyses first included all variables identified in the univariate models and backward selection (*p* ≤ 0.05) was used to generate the final models.

**Results:**

Median age was 39 years (*N* = 866). The proportion of FSWs reporting at least one pregnancy during sex work practice was 16.4%, of whom 42.3% had more than one. Most pregnancies ended with an abortion (67.6%). In multivariate analyses, younger age, longer duration in sex work, previous HIV testing, having a boyfriend and not using condoms with him were significantly (*p* < 0.05) associated with more pregnancies.

**Conclusion:**

One FSW out of five had at least one pregnancy during her sex work practice. Most of those pregnancies, regardless of their origin, ended with an abortion. Improving access to various forms of contraception and safe abortion is the key to reducing unintended pregnancies and consequently, MMM among FSWs in Benin.

## Background

In Sub-Saharan Africa, the maternal mortality ratio (MMR) is the highest in the world with approximately 550 maternal deaths per 100,000 live births as of 2015 [1]. Most of these deaths are avoidable as their main drivers are the lack of access to appropriate quality care [2] and unsafe abortions [3]. Responding to women’s reproductive health needs, such as education on sexual and reproductive health and rights, access to and information on family planning and improved access to quality prenatal, emergency obstetrics, safe abortion and post abortion care, could reduce this burden [4–6]. However, behavioural and structural factors related to sex work, such as violence, sex with multiple partners, inconsistent condom use, stigma and discrimination, increase the risk of poor sexual and reproductive health (SRH) and adverse pregnancy outcomes in the population of female sex workers (FSWs) [5]. Since little is known about pregnancies occurring in FSWs SRH services are limited for this specific group [7].

Because of the lack of empowerment and ability to negotiate condom use [8, 9], as well as the strong economic incentives for multiple partners and the provision of condomless sex [10], FSWs are at high risk of pregnancy. The 12-month overall cumulative incidence is quite high, as observed in Cambodia (20%) and Madagascar (23%) [11, 12]. Unfortunately, these studies did not specify whether FSWs desired those pregnancies or not. Yet this distinction is important since unintended pregnancies are highly associated with numerous negative consequences such as social stigmas, financial burden and unsafe abortion [13].

Worldwide, 40% of all pregnancies are unintended [14] and approximately 50% of these unintended pregnancies end in abortion [15]. Unsafe abortion highly contributes to MMR in developing countries [16]. In some studies, the proportion of unintended pregnancies occurring during sex work was around 90% [13, 17] and lifetime abortion rate varies between 35 and 65% [18–21] among FSWs from low and middle-income countries. With the current available information, it is difficult to know if such abortions mostly occur prior to or after entry into sex work.

Despite the high burden of poor reproductive health, FSWs have a high rate of unmet SRH needs [22, 23] because of the lack of SRH services [24]. A first step to identify those needs is to better quantify this problem and identify the FSWs most at risk. Consequently, this study aimed at gathering knowledge related to pregnancy among FSWs in Benin with the following four objectives: 1) Estimate the frequency of pregnancy occurrence during the practice of sex work; 2) Classify pregnancy outcomes; 3) Assess factors associated with the occurrence of at least one pregnancy during sex work and; 4) Assess factors associated with the occurrence of at least one abortion during the same period among FSWs who became pregnant.

## Methods

**Fig. 1:**
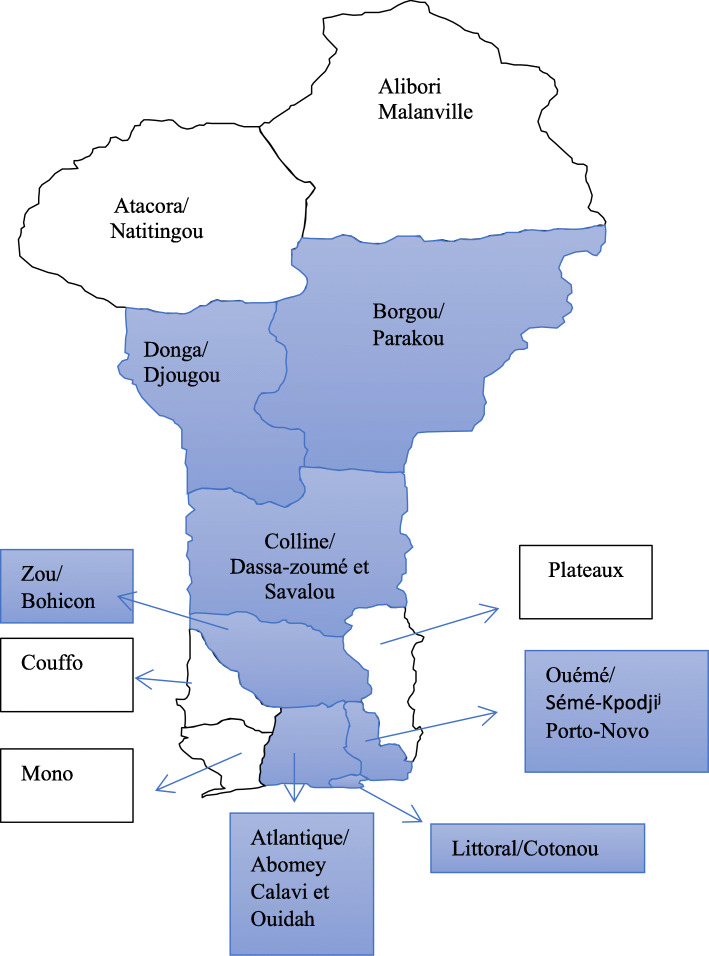
Map of Benin. Blue-colored areas represent the departments and cities of the project. Figure built using an empty map frame freely and openly available at http://www.carte-du-monde.net/pays-1007-carte-benin-vierge.html and modified using Microsoft® Word for Office 365 MSO (16.0.12624.20348) 64-bit, version 2003

We used data from two cross-sectional surveys conducted in 2013 and 2016 that recruited, respectively, 450 and 504 FSWs from numerous sex work sites across the country. The primary objective of these surveys was to describe the overall context of sex work in 11 cities or towns located in seven departments of Benin (Fig. 1) and its evolution over this three-year period, when we implemented an human immunodeficiency viruses (HIV) prevention and reproductive health intervention program aimed at FSWs.

### Data collection

Before the two data collection periods, a local team mapped the different sex work sites in Benin. This mapping allowed an exhaustive census of all important sex work sites in the country and enumerated the FSW population (details given elsewhere) [25].

Then, we used cluster sampling to select a representative sample of sex work sites in the intervention localities (Fig. 1). In a second phase, trained and experienced investigators visited each selected site. All FSWs (defined as women aged ≥18 years and selling sex for money or goods at the time of the study) present at each site were enrolled after having provided informed consent. This process was done in 2013 and 2016 until the projected sample size of at least 450 FSWs was reached for each year. Following the recruitment period, investigators administered a quantitative reproductive health questionnaire during face-to-face interviews with each participant. The same questionnaire was used for both cross-sectional surveys.

### Outcomes

The two outcomes of interest in the present study were the occurrence of at least one pregnancy and that of at least one abortion since the moment each participant started engaging in sex work.

### Independent variables

We explored three types of independent variables during our model selection process: 1) Socio-demographic characteristics (age, region, country of origin, religion, education, marital status, having a boyfriend, cohabitation with a sexual partner, the numbers of dependent individuals and the number of biological children); 2) Sexual behaviours (age at sexual debut, age at first sex work experience, number of years involved in sex work, number of clients during the last working day, number of clients during the last 7 days and money received for the last sexual relation); and 3) Information about the use of SRH prevention services and contraception methods (using at least once SRH prevention services during sex work practice, participating as peer educator in HIV and sexually transmitted infections (STI) preventions activities, being tested for HIV at least once during lifetime, currently using hormonal contraception, condom use with clients and boyfriends in the last 7 days).

### Database merging

We evaluated the impact of merging databases from both surveys (2013 and 2016) as means to enhance the statistical power of our analysis and identified participants that may have contributed information to both surveys, in order to exclude one of their contributions or to consider repeated measures in the data analysis. Because no nominal information was disclosed in both surveys, we used aggregate socio-demographic characteristics to identify potential participants contributing information in both surveys. We explored eight different combinations of six variables stable across time (i.e. month and year of birth, country of origin, religion, education level, age at sexual debut and age at first sex work experience).

### Statistical analyses

Following merger, we carried out descriptive statistics using proportions for discrete variables and means with standard deviations for continuous variables. We then compared the population characteristics between both cross-sectional surveys. Ultimately, we used univariate and multivariate Poisson regression models to identify factors associated with our two outcomes of interest. We estimated adjusted prevalence ratios (aPR) and their 95% confidence intervals (95%CI) with generalized estimating equations (GEE) using a robust variance estimator to decrease the potential impact of a correlation matrix incorrectly specified, and a clustering effect related to the FSWs recruited at the same prostitution site. We also adjusted all the models for survey year (2013 or 2016) to account for potential variations in behavioural characteristics between both surveys. We used a two-step model selection process to choose our independent variables. First, variables associated with the occurrence of at least one pregnancy with *p*-values < 0.2 in the univariate analysis were automatically included in the multivariate model. Then, we removed the least associated variables until all *p*-values were ≤ 0.05. Similar analyses were carried out for the occurrence of abortion among women reporting at least one pregnancy during their sex work practice. We performed all the analyses using SAS 9.4 (SAS Institute, Cary, NC, USA).

### Ethical considerations

To diminish the potential impact of sensitive questions, the interviewers were trained on ethical issues. Each participant provided written informed consent prior to the interview and no nominal information was reported on the questionnaire. The study was approved by the ethics committee of the CHU de Québec – Université Laval (Québec, Canada) and by the National Health Research Ethics Committee in Benin.

## Results

### Database merger

We tested eight different sets of variables in each dataset to validate their specificity. Five of those sets found more than 10 duplicates in the same survey (Additional file 1). Between the three remaining options, we chose the most specific with the fewest variables (options 3 and 8). When we matched the two datasets with the chosen aggregate, we were unable to find any duplicate with option 3 and only two with option 8. Finally, to confirm our decision, we compared all the sites used to recruit FSWs. More than 85% of these sites differed between the two surveys, an observation that also strengthens our choice of merging the two databases together and our confidence in the rarity of duplicates between the two surveys. It is because of that rarity and the uncertainty about the fact it was real duplicates that we decided to leave the data untouched.

### Missing data

Of the 954 participants, 88 (9%) had missing data. Eleven women had missing data for the outcomes of interest related to pregnancy (1%) and 77 (8%) for the independent variables. Participants having missing data for the independent variables were not significantly different with regards to the outcomes, compared to those without [at least one pregnancy, 19.3% vs 16.4% (*p* = 0.48, chi-square); at least one abortion among those who had at least one pregnancy during sex work, 58.8.% vs 67.6% (*p* = 0.47, chi-square)].

### Descriptive analyses

After excluding participants with missing data, our database contained 866 FSWs. Median age was 39 years (interquartile range (IQR): 24–37 years), with the largest proportion of women aged from 20 to 29 years (47.8%). Nearly two-thirds (65.1%) of the women worked in the greater Cotonou region (including the city of Cotonou in Littoral, Abomey-Calavi in Atlantique and Sèmè-Podji in Ouémé), the largest city and economic capital of Benin. Beninese represented 44.0% of the FSWs while Nigerians and Togolese represented, respectively, 26.7 and 18.7% of our sample. Most women had achieved primary (38.1%) or secondary (28.1%) education levels. Regarding marital status, 54.2% of FSWs were divorced or separated and half of them had a boyfriend (52.2%). Only 21.5% of the FSWs were childless and even less in 2016 (15.6% in 2016 vs 27.8% in 2013) (Table 1).

**Table 1:** Sociodemographic characteristics of the studied population (*n* = 866)

Sociodemographic variables	2013 (***n*** = 418)	2016 (***n*** = 448)	Combined population (n = 866)		*p*-value*
	Frequency (%)		Median (IQR)	Frequency (%)	
**Age**			39 (24–37)		
< 20	25 (6.0)	14 (3.1)		39 (4.5)	
20–24	94 (22.5)	87 (19.4)		181 (20.9)	
25–29	113 (27.0)	120 (26.8)		233 (26.9)	
30–34	81 (19.4)	96 (21.4)		132 (15.2)	
35–39	36 (8.6)	35 (7.8)		71 (8.2)	
≥ 40	69 (16.5)	96 (21.4)		165 (19.1)	0.1426
**Region**			–		
Greater Cotonou area	261 (62.5)	303 (67.6)		564 (65.1)	
Regions	157 (37.6)	145 (32.4)		302 (34.9)	0.1090
**Country of origin**			–		
Benin	199 (47.6)	182 (40.6)		381 (44.0)	
Togo	70 (16.8)	92 (20.6)		162 (18.7)	
Nigeria	106 (25.4)	125 (27.9)		231 (26.7)	
Ghana	33 (7.9)	38 (8.5)		71 (8.2)	
Other	10 (2.4)	11 (2.5)		21 (2.4)	0.3223
**Religion**			–		
Catholic	171 (40.9)	194 (43.3)		365 (42.2)	
Other Christian	159 (38.0)	162 (36.2)		321 (37.1)	
Muslim and other	38 (9.1)	50 (11.2)		88 (10.2)	
Traditional	19 (4.6)	23 (5.1)		42 (4.9)	
No religion	31 (7.4)	19 (4.2)		58 (5.8)	0.2540
**Education**			–		
Unschooled	77 (18.4)	87 (19.4)		164 (18.9)	
Primary	160 (38.3)	170 (38.0)		330 (38.1)	
Secondary 1	112 (26.8)	136 (30.4)		248 (28.6)	
Secondary 2 and more	69 (16.5)	55 (12.3)		124 (14.3)	0.2861
**Marital status**			–		
Married	16 (3.8)	19 (4.2)		35 (4.0)	
Divorced or separeted	207 (49.5)	262 (58.5)		469 (54.2)	
Widowed	32 (7.7)	44 (9.8)		76 (8.8)	
Single	163 (39.0)	123 (27.5)		286 (33.0)	**0.0043**
**Has a boyfriend**			–		
Yes	239 (57.2)	213 (47.5)		452 (52.2)	
No	179 (42.8)	235 (52.5)		414 (47.8)	**0.0046**
**Cohabitation with a sexual partner**			–		
Yes	35 (8.4)	65 (14.5)		100 (21.6)	
No	383 (91.6)	383 (85.5)		766 (88.5)	**0.0048**
**Number of dependents**			2 (1–4)		
None	133 (31.8)	64 (14.3)		197 (22.8)	
1 person	55 (13.2)	53 (11.8)		108 (12.5)	
2 persons	59 (14.1)	80 (17.9)		139 (16.1)	
3 persons	58 (13.9)	75 (16.7)		133 (15.4)	
4 persons	38 (9.1)	54 (12.1)		92 (10.6)	
≥ 5 persons	75 (17.9)	122 (27.2)		197 (22.8)	**< 0.0001**
**Number of biological children**			1 (1 - 3)		
None	116 (27.8)	70 (15.6)		186 (21.5)	
1 child	123 (29.4)	126 (28.1)		249 (28.9)	
2 children	83 (19.9)	117 (26.1)		200 (23.1)	
3 children	51 (12.2)	61 (13.6)		112 (12.9)	
≥ 4 children	45 (10.8)	74 (16.5)		119 (13.7)	**< 0.0001**
** Sex work characteristics**					
**Age at first sex**			17 (16–19)		
≤ 15	92 (22.0)	115 (25.7)		207 (23.9)	
16–17	139 (33.3)	120 (26.8)		259 (29.9)	
18–19	127 (30.4)	138 (30.8)		265 (30.6)	
≥ 20	60 (14.4)	75 (16.7)		135 (15.6)	0.1689
**Sex work debut (age)**			24 (20–30)		
≤ 17	29 (6.9)	69 (15.4)		98 (11.3)	
18–21	98 (23.4)	111 (24.8)		209 (24.1)	
22–25	99 (23.7)	108 (24.1)		207 (23.9)	
26–29	69 (16.5)	49 (10.9)		118 (13.6)	
≥ 30	123 (29.4)	111 (24.8)		234 (27.0)	**0.0004**
**Duration in sex work (years)**			4 (2–7)		
≤ 1	105 (25.1)	86 (19.2)		191 (22.1)	
2	83 (19.9)	60 (13.4)		143 (16.5)	
3–4	94 (22.5)	82 (18.3)		176 (20.3)	
5–9	104 (24.9)	97 (21.7)		201 (23.2)	
≥ 10	32 (7.7)	123 (27.5)		155 (17.9)	**< 0.0001**
**Number of clients (last day of work)**			3 (1–4)		
≤ 1	124 (29.7)	102 (22.8)		226 (26.1)	
2 to 3	127 (30.4)	205 (45.8)		332 (38.3)	
4 to 5	100 (23.9)	89 (19.9)		189 (21.8)	
≥ 5	67 (16.0)	52 (11.6)		119 (13.7)	**< 0.0001**
**Number of clients (last 7 days)**			13 (6–25)		
≤ 5	103 (24.6)	100 (22.3)		203 (23.4)	
6–10	55 (13.2)	114 (25.5)		169 (19.5)	
11–15	62 (14.8)	81 (18.1)		143 (16.5)	
16–20	77 (18.4)	73 (16.3)		150 (17.3)	
≥ 20	121 (29.0)	80 (17.9)		201 (23.2)	**< 0.0001**
**Money received for last sexual relation (FCFA)**^a^			2500 (1500–5000)		
≤ 1500	137 (32.8)	120 (26.8)		257 (29.7)	
1501–2000	83 (19.9)	90 (20.1)		173 (20.0)	
2001–5000	137 (32.8)	157 (35.0)		294 (34.0)	
> 5000	61 (14.6)	81 (18.1)		142 (16.4)	0.2078
** Prevention services**					
**Use at least one SRH prevention services during sex work**			–		
Yes	110 (26.3)	215 (48.0)		325 (37.5)	
No	308 (73.7)	233 (52.0)		541 (62.5)	**< 0.0001**
**Participates as peer worker in HIV and STI prevention activities**			–		
Yes	29 (6.9)	62 (13.8)		91 (10.5)	
No	389 (93.1)	386 (86.2)		775 (89.5)	** 0.0003**
**Tested for HIV at least once during lifetime**			–		
Yes	364 (87.1)	436 (97.3)		800 (92.4)	
No	54 (12.9)	12 (2.7)		66 (7.6)	**0.0217**
** Contraception**					
**Currently using hormonal contraception**			–		
Yes	75 (17.9)	92 (20.5)		167 (19.3)	
No	343 (82.1)	356 (79.5)		699 (80.7)	0.3338
**Condom use (the last 7 days)**					
With clients			–		
Not always	41 (9.8)	35 (7.8)		76 (8.8)	
Always	377 (90.1)	413 (92.2)		790 (91.2)	0.2995
With non-paying partners			–		
Never/Not always	107 (25.6)	93 (20.8)		200 (23.1)	
Always	42 (10.1)	23 (5.1)		65 (7.5)	
No sexual relation	269 (64.4)	332 (74.1)		601 (69.4)	**0.0023**

^*^ According to chi-square comparing the frequencies between 2013 and 2016

^a^ FCFA (1 US dollars ± = 500 FCFA)

Median age at sexual debut was 17 years (IQR: 16–19), whereas median age when starting involvement in sex work was 24 years (IQR: 20–30). Median duration in sex work was 4 years (IQR: 2–7). The median number of clients in the last week was 13 (IQR: 6–25) and the last sexual transaction brought back an average of approximately five US dollars (2500 (*Franc des communautés financières africaines*) FCFA). FSWs surveyed in 2016 had been involved in sex work for a longer period as compared to those surveyed in 2013 (≥ 10 years as sex workers, 27.5% vs 7.7% in 2013) and had fewer clients (Table 1).

The overall use of SRH services was higher in 2016 compared to 2013 and most participants had ever been tested for HIV (97.3% in 2016 vs 87.1% in 2013). In the combined data, the use of SRH services was less common (37.5%) as was the use of hormonal contraception (19.3%). Finally, consistent condom use was high with clients (91.2%) but not with boyfriends (24.5%, 65/265) (Table 1).

### Pregnancies among female sex workers

The proportion of women with at least one pregnancy since sex work initiation was 16.4% (142/866) using the merged dataset while it was 18.2 and 14.7%, for the 2013 and 2016 surveys, respectively (*p* = 0.17, chi-square). Of all the women who had at least one pregnancy occurring while being a sex worker, 42.3% (60/142) had more than one (mean 1.78, SD 1.2). In addition, most FSWs (87%) declared that the pregnancy originated from their boyfriends (vs 13% from the clients) (Table 2).

**Table 2:** Overview of sex workers’ pregnancies during sex work practice

	2013 (n = 418)	2016 (n = 448)	*p*-value*	Combined population (***n*** = 866)			
	Frequency (%)			Mean (sd)	Frequency (%)		
**At least one pregnancy**							
Yes	76 (18.2)	66 (14.7)			142 (16.4)		
No	342 (81.8)	382 (85.3)	0.1707		724 (83.6)		
**More than one pregnancy**							
Yes	35 (46.1)	25 (37.9)			60 (42.3)		
No	41 (53.9)	41 (62.1)			82 (57.8)		
**At least one abortion**							
Yes	55 (73.3)	41 (63.1)			96 (68.6)		
No	20 (26.7)	24 (36.9)	0.1931		44 (31.4)		
**More than one abortion**							
Yes	32 (58.2)	22 (53.7)			54 (56.3)		
No	23 (41.8)	19 (46.3)			42 (43.8)		
**Number of pregnancies**							
1	41 (54.0)	41 (62.1)			82 (57.8)		
2	19 (25.0)	12 (18.2)			31 (21.8)		
3	9 (11.8)	6 (9.1)			15 (10.6)		
4	6 (7.9)	4 (6.1)			10 (7.2)		
5	–	1 (1.5)			1 (0.7)		
6	–	2 (3.0)			2 (1.4)		
7	1 (1.3)				1 (0.7)		
** Number of pregnancies**							
**Pregnancy outcomes**						**Boyfriend**	**Clients**
Live birth					60 (23.7)	54 (24.8)	6 (18.2)
Stillbirth					7 (2.8)	5 (2.3)	2 (6.1)
Miscarriage					19 (7.5)	16 (7.3)	3 (9.1)
Abortion				1.63 (1.1)	165 (65.2)	143 (65.6)	22 (66.7)
**Total number of pregnancies**^a^				1.78 (1.2)	253 (100.0)^a^	220 (87.0)^a^	33 (13.0)

* According to chi-square

^a^ Two FSWs were pregnant during data collection and thus had missing values for pregnancy outcome

### Pregnancy outcomes in female sex workers

Of the 142 women who had at least one pregnancy since sex work initiation, 67.6% (96/142) had at least one abortion and 43.7% (42/96) of the latter had more than one. This proportion was slightly lower in 2016 (62.1%) than in 2013 (72.4%), but the difference was not statistically significant (*p* = 0.19, chi-square). The proportion of all 253 pregnancies that occurred in 142 women that ended in abortion was similar among women reporting getting pregnant from a boyfriend (65.6%) and those reporting being pregnant from a client (66.7%) (Table 2).

### Factors associated with the occurrence of at least one pregnancy during sex work

Table 3 displays the multivariate analysis of the factors associated with the occurrence of pregnancies during sex work (see Table 2 in the additional files for the crude frequencies and univariate analysis). In multivariate analysis, the risk of having a pregnancy during the practice of sex work decreased as women got older (*p* < 0.0001). We observed the opposite trend with the number of years women were involved in sex work (*p* < 0.0001). Having a boyfriend was associated with a 70% increase in the occurrence of at least one pregnancy whereas consistent condom use with boyfriends had a protective effect (aPR = 0.55, 95%CI: 0.3–0.9). Women from Togo and Nigeria were 30% more likely to have had at least a pregnancy compared to FSWs from Benin (aPR = 1.28, 95%CI: 0.9–1.8). Finally, women who had tested for HIV during their lifetime reported being pregnant at least once during sex work more often (aPR = 3.74, 95%CI: 1.5–9.2) than the few women who had not (Table 3).

**Table 3:** Multivariate analysis for the risk of having at least one pregnancy during sex work (n = 866)

Sociodemographic characteristics	aPR	95% CI	*P-*value*	*P*-trend**
**Age**				
< 20	1			
20–24	0.91	0.4–1.7		
25–29	0.71	0.3–1.5		
30–34	0.32	0.2–0.7		
35–39	0.26	0.1–0.7		
≥ 40	0.29	0.1–0.7	**< 0.0001**	**< 0.0001**
**Country of origin**				
Benin	1			
Ghana	0.83	0.4–1.7		
Togo	1.28	0.9–1.8		
Nigeria	1.29	0.9–1.8		
Other	2.42	1.3–4.5	**0.0343**	**–**
**Has a boyfriend**				
No	1			
Yes	1.74	1.1–2.8	**0.0246**	–
**Sexual behaviors**				
**Duration in sex work (years)**				
≤ 1	1			
2	1.45	0.8–2.6		
3–4	2.22	1.4–3.5		
5–9	2.94	1.9–4.6		
≥ 10	4.01	2.0–7.9	**< 0.0001**	**< 0.0001**
**Prevention services**				
**HIV testing at least once during lifetime**				
No	1			
Yes	3.74	1.–9.2	**0.0040**	
**Contraception**				
**Condom use (the last 7 days)**				
With non-paying partners				
Never/ Not always	1			
Always	0.55	0.3–0.9		
No sexual relation	0.60	0.4–0.9	**0.0069**	

* *p*-value in the multivariate analysis, adjusted for the year of the two different surveys; *p*-values written in bold are ≤0.05

** *p-value, test for linear trend in the multivariate analysis,* adjusted for the year of the two different surveys; *p*-values written in bold are ≤0.05

*° In FCF*A (1 US dollars ± = 500 FCFA)

### Factors associated with at least one abortion among women who became pregnant during sex work

Out of the 142 women who reported at least one pregnancy during sex work, 140 specified their pregnancies outcomes and the multivariate analysis showed that there was an overall significant association between age and the likelihood of having had at least one abortion, but no significant trend (Table 4). FSWs from Togo were more likely to have had at least one abortion compared to those from Benin (aPR = 1.31, 95%CI: 1.0–1.7) and there was an overall significant difference in the likelihood of having had at least an abortion according to the country of origin (*p* = 0.0047). Increasing numbers of children decreased the number of abortion (p-trend = 0.0049). However, the association was in the opposite direction for the number of dependents, but with no significant trend. Among FSWs who had to take care financially of one other person, there was a 1.3-fold increase in the likelihood of having an abortion (aPR = 1.32, 95%CI: 1.0–1.8) compared to women with no dependent. A similar association was observed for the fact of having five dependents (aPR = 1.28, 95%CI: 1.0–1.6), but no significant associations were found for women having two, three or four dependents. Consistent condom use, both with clients (aPR = 0.71, 95%CI: 0.5–1.0) and boyfriends (aPR = 0.61 95%CI: 0.4–0.9), was protective against abortion (Table 4). As for the factors associated with the occurrence of at least one pregnancy during sex work practice, the frequencies and univariate analysis are available in the additional files (Table 3).

**Table 4:** Multivariate analysis for the risk of having at least one abortion for women who had at least one pregnancy during sex work (n = 140)

Sociodemographic characteristics	aPR	95% CI	*P-*value*	P-trend **
**Age**				
< 20	1			
20–24	1.30	0.7–2.5		
25–29	1.14	0.6–2.3		
30–34	1.85	0.9–3.9		
35–39	1.02	0.3–3.2		
≥ 40	1.83	0.8–4.0	**0.0252**	0.7898
**Country of origin**				
Benin	1			
Ghana	0.97	0.7–1.4		
Togo	1.31	1.0–1.7		
Nigeria	1.08	0.6–1.4		
Other	0.49	0.2–1.3	**0.0047**	
**Number of dependents**				
None	1			
1 person	1.32	1.0–1.8		
2 persons	1.08	0.8–1.5		
3 persons	1.13	0.9–1.5		
4 persons	1.08	0.6–1.3		
≥ 5 persons	1.28	1.0–1.6	**0.0084**	0.2776
**Number of biological children**				
None	1			
1 child	0.61	0.5–0.8		
2 children	0.68	0.5–0.9		
3 children	0.56	0.4–0.8		
≥ 4 children	0.73	0.5–1.0	**0.0008**	**0.0049**
**Contraception**				
**Condom use (the last 7 days)**				
With clients				
Not always	1			
Always	0.71	0.5–1.0	**0.0415**	–
With non-paying partners				
Never/ Not always	1			
Always	0.61	0.4–0.9		
No sexual relation	0.86	0.7–1.1	**0.0021**	–

* *p*-value in the multivariate analysis, adjusted for the year of the two different surveys; *p*-values written in bold are ≤0.05

** *p*-*value, test for linear trend in the multivariate analysis,* adjusted for the year of the two different surveys; *p*-values written in bold are ≤0.05

*° In FCF*A (1 US dollars ± = 500

## Discussion

Globally, FSWs are at high risk of unplanned pregnancy and its adverse consequences, including death; Benin is no exception. Our study is one of the first in West Africa that attempts to quantify this risk in order to develop programs that meet FSWs’ SRH needs.

The proportion of women with at least one pregnancy during sex work practice is relatively high in Benin and most of those pregnancies ended in abortion. Young immigrant women, those practicing sex work for longer periods (more than 2 years) and those having a boyfriend are the most at risk for pregnancy. The ones who always use condoms with their boyfriends have significantly fewer pregnancies and abortion, but this represents only a minority of women. The older the women get and the more biological children they have the less they have an abortion to end their pregnancy.

Our findings indicate that a minimum of 16% of the FSWs had at least one pregnancy during their sex work practice and almost half of the latter (42%) had more than one, with an average of 1.8 pregnancies per women. This proportion of women who got pregnant during sex work is similar to the one observed in other studies (around 20%) [11, 12, 26–28]. Most of the women were of reproductive age between 20 and 29 years old and most of their pregnancies came from their boyfriends. Clearly less stigma is associated with having a pregnancy from a boyfriend than from a client. Furthermore, condom use with boyfriends was low as reported elsewhere [29]. The non-use of condoms with boyfriends helps the FSWs to make a distinction between their personal and professional life [30]. In addition, the non-use of condoms is usually at the boyfriend’s request and is a way to prove the fidelity of the FSWs [31]. All these perceptions make us believe that a part (even though small) of those pregnancies might be intended, especially when considering that motherhood is highly valued in African countries and is a way for FSWs to gain respect in their community [32]. However, this is worrisome since FSWs’ boyfriends are at high risk of HIV and STI [33].

FSWs who had been tested for HIV at least once during their lifetime reported at least one pregnancy during sex work almost 4 times more often than the ones who never did. We observed this disparity even though almost all FSWs had been tested for HIV (92%). The first hypothesis for explaining this observation is that pregnant women are systematically tested for HIV when seeking prenatal care [34]. The second hypothesis is that women who have unprotected sex consider themselves more likely to contract HIV and this risk perception explains why they had been tested [25]. In Benin, FSWs are overly represented in the HIV epidemic [35] with an HIV prevalence of nearly 20% in the FSW population [36] and a non optimal adherence to antiretroviral therapy (ART) [37]. This explains why the majority of the services available to prevent HIV among FSWs in Sub-Saharan Africa focuses on the promotion and the delivery of condoms instead of focussing on SRH in general [24]. HIV testing services could however be used as a good opportunity to discuss and integrate broader SRH needs, including contraception [22].

FSWs aged < 20 years were particularly at risk of getting pregnant. Indeed, younger FSWs may be less experienced with condom negotiation and have more unprotected sex with their non-paying partners [38]. Those two behaviors put them at higher risk for pregnancy. Research in Cambodia found that younger FSWs were at higher risk for pregnancy and that their ability to negotiate condom use is critical to prevent pregnancy [11]. Since condom negotiation is often difficult, especially for younger sex workers, and condom breakage is common [39, 40], the use of dual contraception is the best method to prevent pregnancy [12]. FSWs in Sub-Saharan Africa underuse hormonal and dual contraception [21, 41–43]. We observed the same situation in our sample where only 48% of the women ever used SRH services during sex work and only 20% were currently using hormonal contraception. In Benin’s general population the use of modern contraception is low. The government promotes family planning as part of its national health plan [44] since the best way to prevent maternal and newborn death is the provision of modern contraception combined with adequate care for pregnant women [45]. Many barriers are associated with the use of modern contraception. One of the most frequent barriers is the fear of side effects associated with modern contraception and the opposition toward it [46]. FSWs face numerous stigmas when they need to access health care [47]. They need to have a special attention to access to modern contraception and family planning services. Those services need to focus, among others, on young women who are likely to spend more years as sex workers.

In Benin, the abortion law allows women to access that procedure only if the life of the mother is in danger, in cases of rape, incest or malformations of the foetus [48]. Abortion in Cotonou seems to be fairly accessible in small private health centers but its cost (between 25.50 and 89.00 US dollars) [49] appears highly prohibitive for FSWs. Regardless of origin, 65% of all pregnancies occurring during sex work practice end in abortion. This abortion rate is in the upper range in comparison with previous observations (between 30 and 65%) even if previous studies generally reported lifetime abortion rates among FSWs [18–22, 41]. The high abortion rate likely reflects a high rate of unsafe abortion procedures, which in low resource settings is known to contribute to 13% of overall maternal mortality [3].

With the information available, we did not know in what kind of settings the FSWs accessed abortion. We do know that FSWs have limited economic options, low education and many dependents [50] and those factors place them at higher risk of unsafe abortion [51–53].

The high abortion rate observed here might be explained by the fact that the vast majority of FSWs already had their children prior to their involvement in sex work and the number of children is a determining factor for abortion [54, 55]. As we could observe in our sample, FSWs who had more dependents had more chance of having an abortion. Financial vulnerabilities often lead women to become sex workers [56] and having a child increases that financial burden [13]. However, FSWs who have more children have fewer abortions. This surprising result can be explained by the type of study we used. A cross-sectional survey does not enable us to determine the temporality of the events, meaning that maybe the non-use of abortion caused the FSWs to have more children and that fact can explain why the number of children appeared as a protective factor.

Older age is usually a factor associated with abortion [19, 55, 57, 58]. In our analysis, we could not observe a clear trend as we did for the association between age and pregnancy occurrence. We could only use the subsample of 140 women who reported at least one pregnancy during sex work to identify the factors associated with abortion. This relatively small sample size could explain the difficulty to identify clear trends. In our population, as in many countries and populations, abortion was associated with age, marital status and economic factors [45].

### Strengths and limitations

We decided to merge the two databases to enhance our statistical power. To do so, we made sure that the data collection followed the identical process and the same tools. We verified that the outcomes of interest had almost equivalent prevalence. However, a study carried out in Cotonou (data not published), showed that FSWs stayed on average 1 year in that city. Knowing that, we could deduce that after 3 years 12.5% of the FSWs would still be in each location. The possibility that we could not identify potential FSWs who would have answered our survey in 2013 and 2016 could affect our estimates. Indeed, without changing the number of observations, the presence of duplicate cases would reduce the effective sample size and the precision of the estimates [59]. To reduce this risk, we compared the two samples to identify possible duplicates and found only two, thus suggesting this would concern only a few women and would thus have minimal impact on the validity of our analyses.

In addition, we could have underestimated the frequency of our outcomes since pregnancy and abortion are sensitive topics. There is a high probability that some women did not disclose some pregnancies or abortions. Consequently, the frequency of pregnancies and abortions that we observed are likely to be underestimated.

Because we used a cross-sectional design, we cannot asses the temporality of the factors associated with pregnancy and abortion. Moreover, it is not possible to establish a causal link between these factors versus pregnancy and abortion and we do not know if the pregnancies were wanted or not. In the future, a longitudinal study could address those limits.

On the positive side, the sample is representative of FSWs from large cities (Cotonou, Porto Novo) and much smaller towns in Benin. Usually the studies assessing pregnancy are only from urban settings [57]. The scale of the survey allowed a good representativeness of the FSW population. Furthermore, this is the first study to assess pregnancy and abortion over the sex work practice period. Most studies exploring these issues cover a specific time period or the entire women’s lifetime [57]. Lastly, the large sample size gave us the opportunity to consider several potential factors associated with the occurrence of pregnancies during sex work.

## Conclusion

To conclude, one FSW out of five had at least one pregnancy during her sex work practice. Most of those pregnancies, regardless of their origin, ended with an abortion. Our results suggest that prevention services need to continue to promote condom use and that dual protection is the key to reduce unintended pregnancies in the FSW population. By doing so, maternal mortality could decrease in this vulnerable population. With the available data, this study was not able to specify whether those pregnancies were wanted or not and in which conditions women underwent abortion. More research is needed to find answers to these questions.

## Supplementary information

**Additional file 1.** Duplicate observations identification test.

**Additional file 2 **Univariate analysis for the risk of having at least one pregnancy during sex work (*n* = 866).

**Additional file 3 **Univariate analysis for the risk of having at least one abortion for women who had at least one pregnancy during sex work (*n* = 140).

## Data Availability

The datasets used and analysed during the current study are available from the corresponding author an reasonable request.

## Abbreviations

aPR: Adjusted proportion ratio
ART: Antiretroviral therapy
FCFA: Franc des communautés financières africaines
FSWs: Females sex workers
GEE: Generalized estimating equations
HIV: Human immunodeficiency viruses
MMM: Maternal mortality and morbidity
MMR: Maternal mortality ratio
SD: Standard deviation
SRH: Sexual and reproductive health
STI: Sexually transmitted infections
