# Supplementary material for: Overview and factors associated with pregnancies and abortions occurring in sex workers in Benin
Source: BMC Womens Health. 2020 Nov 9;20:248. doi: 10.1186/s12905-020-01091-6 (PMC7650197; doi:10.1186/s12905-020-01091-6)
Supplement: Supplementary file 1 — Additional file 1. Duplicate observations identification test. [file 12905_2020_1091_MOESM1_ESM.docx]

|  |  | Number of variables | Number of identical women found in a same survey | | | | | | Number of identical women found between surveys |
| --- | --- | --- | --- | --- | --- | --- | --- | --- | --- |
| Option | Variable set |  | 2013 survey | | | 2016 survey | | |  |
|  |  |  | 2 women | 3 women | ≥4 women | 2 women | 3 women | ≥4 women |  |
| 1 | Country of origin, religion, education, age at first sex and age at sex work debut | 5 | 20 | 2 | 1 | 13 | 2 | 0 |  |
| 2 | Year of birth, month of birth, country of origin, religion and education | 5 | 16 | 1 | 0 | 16 | 4 | 10 |  |
| **3*** | Year of birth, month of birth, country of origin, religion, education and age at sex work debut | 6 | 2 | 0 | 0 | 2 | 3 | 0 | 0 |
| 4 | Year of birth, month of birth, country of origin, religion, education and age at first sex | 6 | 4 | 0 | 0 | 21 | 1 | 0 |  |
| 5 | Year of birth, month of birth, country of origin, religion, education, age at first sex and age at sex work debut | 7 | 1 | 0 | 0 | 1 | 0 | 0 |  |
| 6 | Year of birth, country of origin, religion, education and age at sex work debut (3- month of birth) | 5 | 15 | 1 | 0 | 16 | 3 | 0 |  |
| 7 | Year of birth, country of origin, religion, education and age at first sex (4 - month of birth) | 5 | 17 | 1 | 1 | 30 | 3 | 1 |  |
| **8*** | Year of birth, country of origin, religion, education, age at first sex and age at sex work debut (5 - month of birth) | 6 | 3 | 0 | 0 | 5 | 0 | 0 | 2 |

**Additional file 1 - Duplicate observations identification test**

* Option prioritized, less variables than option 5 and the less false duplicate.
