# Supplementary material for: Overview and factors associated with pregnancies and abortions occurring in sex workers in Benin
Source: BMC Womens Health. 2020 Nov 9;20:248. doi: 10.1186/s12905-020-01091-6 (PMC7650197; doi:10.1186/s12905-020-01091-6)
Supplement: Supplementary file 2 — Additional file 2 Univariate analysis for the risk of having at least one pregnancy during sex work (n = 866). [file 12905_2020_1091_MOESM2_ESM.docx]

**Additional file 2 - Univariate analysis for the risk of having at least one pregnancy during sex work (n = 866)**

|  | Proportion pregnant* | PR | 95% CI | P-value** |
| --- | --- | --- | --- | --- |
| **Sociodemographic characteristics** |  |  |  |  |
| **Age** |  |  |  |  |
| < 20  20 - 24  25 - 29  30 - 34  35 - 39  ≥ 40 | 6/39 (15.9) | 1  1.51  1.48  0.73  0.55  0.59 | 0.8 - 2.9  0.7 - 3.3  0.3 - 1.8  0.2 - 1.5  0.2 - 1.4 |  |
|  | 42/181 (23.2) |  |  |  |
|  | 53/233 (22.8) |  |  |  |
|  | 20/177 (11.3) |  |  |  |
|  | 6/71 (8.5) |  |  |  |
|  | 15/165 (9.1) |  |  | **<0.0001** |
| **Region** |  |  |  |  |
| Greater Cotonou area | 105/564 (18.6) | 1 |  |  |
| Regions | 37/302 (12.3) | 0.66 | 0.4 - 1.1 | **0.1294** |
| **Country of origin** |  |  |  |  |
| Benin | 58/381 (15.2) | 1 |  |  |
| Ghana | 7/71 (9.9) | 0.65 | 0.4 - 1.3 |  |
| Togo  Nigeria | 33/162 (20.4) | 1.34  1.08 | 0.9 - 1.9  0.7 - 1.6 |  |
|  | 38/231 (16.5) |  |  |  |
| Other | 6/21 (28.6) | 1.88 | 1.1 - 3.3 | **0.0751** |
| **Religion** |  |  |  |  |
| Catholic | 58/365 (15.9) | 1 |  |  |
| Other Christian | 53/321 (16.5) | 1.04 | 0.8 - 1.4 |  |
| Muslim and others | 18/88 (20.5) | 1.29 | 0.8 - 2.0 |  |
| Traditional | 8/42 (19.1) | 1.20 | 0.7 - 2.1 |  |
| No religion | 5/50 (10.0) | 0.63 | 0.2 - 1.7 | 0.7101 |
| **Education** |  |  |  |  |
| Unschooled | 21/164 (12.8) | 1 |  |  |
| Primary | 58/330 (17.6) | 1.37 | 0.9 - 2.3 |  |
| Secondary 1 | 37/248 (14.9) | 1.17 | 0.8 - 1.8 |  |
| Secondary 2 and more | 26/124 (21.0) | 1.64 | 1.0 - 2.8 | 0.2037 |
| **Marital status** |  |  |  |  |
| Married | 11/35 (31.4) | 1 |  |  |
| Divorced or separated | 74/469 (15.8) | 0.50 | 0.3 - 0.9 |  |
| Widowed | 6/76 (7.9) | 0.25 | 0.1 - 0.6 |  |
| Single | 51/286 (17.8) | 0.57 | 0.3 - 1.0 | **0.0017** |
| **Has a boyfriend** |  |  |  |  |
| No | 37/414 (8.9) | 1 |  |  |
| Yes | 105/452 (23.2) | 2.60 | 1.8 - 3.7 | **< 0.0001** |
| **Cohabitation with a sexual partner** |  |  |  |  |
| No | 111/766 (14.5) | 1 |  |  |
| Yes | 31/100 (31.0) | 2.14 | 1.4 - 3.2 | **0.0003** |
| **Number of dependents** |  |  |  |  |
| None | 29/197 (14.7) | 1 |  |  |
| 1 person | 19/108 (17.6) | 1.20 | 0.5 - 2.6 |  |
| 2 persons | 30/139 (21.6) | 1.47 | 1.0 - 2.3 |  |
| 3 persons | 21/133 (15.8) | 1.07 | 0.6 - 1.9 |  |
| 4 persons | 20/92 (21.7) | 1.48 | 0.8 - 2.7 |  |
| ≥ 5 persons | 23/197 (11.7) | 0.79 | 0.5 - 1.4 | 0.3164 |
| **Number of biological children** |  |  |  |  |
| None | 33/186 (17.7) | 1 |  |  |
| 1 child | 53/249 (21.3) | 1.20 | 0.8 - 1.9 |  |
| 2 children | 30/200 (15.0) | 0.85 | 0.5 - 1.4 |  |
| 3 children | 13/112 (11.6) | 0.65 | 0.3 - 1.3 |  |
| ≥ 4 children | 13/119 (10.9) | 0.62 | 0.3 - 1.2 | **0.0231** |
| **Sexual behaviors** |  |  |  |  |
| **Age at first sex** |  |  |  |  |
| ≤ 15 | 50/207 (24.1) | 1 |  |  |
| 16 - 17 | 42/259 (16.2) | 0.67 | 0.5 - 1.0 |  |
| 18 - 19 | 35/265 (13.2) | 0.55 | 0.4 - 0.8 |  |
| ≥ 20 | 15/135 (11.1) | 0.46 | 0.3 - 0.8 | **0.0032** |
| **Sex work debut (age)** |  |  |  |  |
| ≤ 17 | 22/98 (22.45) | 1 |  |  |
| 18 - 21 | 47/209 (22.49) | 1.00 | 0.6 - 1.6 |  |
| 22 - 25 | 41/207 (19.81) | 0.88 | 0.5 - 1.5 |  |
| 26 - 29 | 17/118 (14.41) | 0.64 | 0.4 - 1.1 |  |
| ≥ 30 | 15/234 (6.41) | 0.29 | 0.2 - 0.5 | **<0.0001** |
| **Duration in sex work (years)** |  |  |  |  |
| ≤ 1 | 17/191 (8.90) | 1 |  |  |
| 2 | 21/143 (14.69) | 1.65 | 0.9 - 3.1 |  |
| 3 - 4 | 35/176 (19.89) | 2.23 | 1.5 - 3.4 |  |
| 5 - 9 | 44/201 (21.89) | 2.46 | 1.7 - 3.6 |  |
| ≥ 10 | 25/155 (16.13) | 1.81 | 0.9 - 3.6 | **<0.0001** |
| **Number of clients (last day of work)** |  |  |  |  |
| ≤ 1 | 43/226 (19.47) | 1 |  |  |
| 2 to 3 | 53/332 (15.96) | 0.82 | 0.6 - 1.2 |  |
| 4 to 5 | 26/189 (13.76) | 0.71 | 0.5 - 1.1 |  |
| ≥ 5 | 19/119 (15.97) | 0.82 | 0.5 - 1.3 | 0.4605 |
| **Number of clients (last seven days)** |  |  |  |  |
| ≤ 5 | 44/203 (21.67) | 1 |  |  |
| 6 - 10 | 21/169 (12.43) | 0.57 | 0.4 - 0.9 |  |
| 11 - 15 | 19/143 (13.29) | 0.61 | 0.4 - 1.0 |  |
| 16 - 20 | 26/150 (17.33) | 0.80 | 0.5 - 1.2 |  |
| ≥ 20 | 32/201 (15.92) | 0.73 | 0.5 - 1.1 | **0.1272** |
| **Money received for last sexual relation °** |  |  |  |  |
| ≤ 1500 | 25/257 (9.73) | 1 |  |  |
| 1501 - 2000 | 30/173 (17.34) | 1.78 | 1.0 - 3.2 |  |
| 2001 - 5000 | 56/294 (19.05) | 1.96 | 1.2 - 3.1 |  |
| > 5000 | 31/142 (21.83) | 2.24 | 1.3 - 4.0 | **0.0210** |
| **Prevention services** |  |  |  |  |
| **Use at least one SRH prevention services during sex work** | |  |  |  |
| No | 82/541 (15.16) | 1 | 0.9 - 1.6 |  |
| Yes | 60/325 (18.46) | 1.22 |  | 0.2024 |
| **Participate as peer worker in HIV and STI prevention activities** | |  |  |  |
| No | 129/775 (16.65) | 1 | 0.5 - 1.6 |  |
| Yes | 13/91 (14.29) | 0.86 |  | 0.6156 |
| **HIV testing at least once during lifetime** | |  |  |  |
| No | 4/66 (6.06) | 1 |  |  |
| Yes | 138/800 (17.25) | 2.85 | 1.3 - 6.2 | **0.0090** |
| **Contraception** |  |  |  |  |
| **Currently using hormonal contraception** | |  |  |  |
| No | 105/699 (15.02) | 1 |  |  |
| Yes | 37/167 (22.16) | 1.47 | 1.0 - 2.2 | **0.0540** |
| **Condom use (last seven days)** | |  |  |  |
| With clients | |  |  |  |
| Not always | 11/73 (15.07) | 1 |  |  |
| Always | 131/790 (16.59) | 1.10 | 0.6 - 2.0 | 0.7595 |
| With non-paying partners | |  |  |  |
| Never/ Not always | 61/200 (30.50) | 1 |  |  |
| Always | 11/65 (16.92) | 0.55 | 0.3 - 1.0 |  |
| No sexual relation | 70/601 (21.83) | 0.38 | 0.3 - 0.5 | **<0.0001** |

*Proportion of women with at least one pregnancy during sex work

** p-value in the univariate analysis. P-values written in bold are <0.20

*** p-value in the multivariate analysis, adjusted for the year of the two different surveys; p-values written in bold are ≤0.05

**** *p-value, test for linear trend in the multivariate analysis,* adjusted for the year of the two different surveys; p-values written in bold are ≤0.05

*° In FCF*A (1 US dollars $\pm$= 500 FCFA)
