# Supplementary material for: Overview and factors associated with pregnancies and abortions occurring in sex workers in Benin
Source: BMC Womens Health. 2020 Nov 9;20:248. doi: 10.1186/s12905-020-01091-6 (PMC7650197; doi:10.1186/s12905-020-01091-6)
Supplement: Supplementary file 3 — Additional file 3 Univariate analysis for the risk of having at least one abortion for women who had at least one pregnancy during sex work (n = 140). [file 12905_2020_1091_MOESM3_ESM.docx]

**Additional file 3 - Univariate analysis for the risk of having at least one abortion for women who had at least one pregnancy during sex work** **(n = 140)**

|  | Prevalence | PR | 95% CI | P-value* |
| --- | --- | --- | --- | --- |
| **Sociodemographic characteristics** |  |  |  |  |
| **Age** |  |  |  |  |
| < 20  20 - 24  25 - 29  30 - 34  35 - 39  ≥ 40 | 3/6 (50.0) | 1 |  |  |
|  | 29/41 (70.7) | 1.41 | 0.6 - 3.2 |  |
|  | 31/53 (58.5) | 1.17 | 0.5 - 2.7 |  |
|  | 17/19 (89.5) | 1.79 | 0.8 - 4.1 |  |
|  | 3/6 (50.0) | 1.00 | 0.4 - 2.8 |  |
|  | 13/15 (86.7) | 1.73 | 0.8 - 4.0 | **0.0154** |
| **Region** |  |  |  |  |
| Greater Cotonou area | 73/104 (70,2) | 1 |  |  |
| Regions | 23/36 (63.9) | 0.91 | 0.7 - 1.2 | 0.4479 |
| **Country of origin** |  |  |  |  |
| Benin | 37/58 (63.8) | 1 |  |  |
| Ghana | 5/7 (71.4) | 1.12 | 0.8 - 1.6 |  |
| Togo  Nigeria | 25/33 (75.8) | 1.19  1.18 | 1.0 - 1.4  1.0 - 1.4 |  |
|  | 27/36 (75.0) |  |  |  |
| Other | 2/6 (33.3) | 0.52 | 0.2 - 1.6 | **0.1188** |
| **Religion** |  |  |  |  |
| Catholic | 41/57 (71.9) | 1 |  |  |
| Other Christian | 35/53 (66.0) | 0.92 | 0.7 - 1.1 |  |
| Muslim and others | 11/18 (61.1) | 0.85 | 0.6 - 1.3 |  |
| Traditional | 7/8 (87.5) | 1.22 | 1.0 - 1.7 |  |
| No religion | 2/4 (50.0) | 0.70 | 0.2 - 1.9 | 0.4061 |
| **Education** |  |  |  |  |
| Unschooled | 12/21 (57.1) | 1 |  |  |
| Primary | 38/56 (67.9) | 1.19 | 0.8 - 1.9 |  |
| Secondary 1 | 27/37 (73.0) | 1.28 | 0.8 - 2.0 |  |
| Secondary 2 and more | 19/26 (73.1) | 1.28 | 0.8 - 2.0 | 0.6514 |
| **Marital status** |  |  |  |  |
| Married | 5/10 (50.5) | 1 |  |  |
| Divorced or separated | 53/73 (72.6) | 1.45 | 0.7 - 3.0 |  |
| Widowed | 3/6 (50.0) | 1.00 | 0.3 - 3.2 |  |
| Single | 35/51 (68.6) | 1.37 | 0.6 - 3.1 | 0.4783 |
| **Has a boyfriend** |  |  |  |  |
| No | 22/36 (61.1) | 1 |  |  |
| Yes | 74/104 (71.2) | 1.16 | 0.9 - 1.6 | 0.3403 |
| **Cohabitation with a sexual partner** |  |  |  |  |
| No | 78/110 (71.0) | 1 |  |  |
| Yes | 18/30 (60.0) | 0.85 | 0.6 - 1.3 | 0.4018 |
| **Number of dependents** |  |  |  |  |
| None | 20/28 (71.4) | 1 |  |  |
| 1 person | 13/19 (68.4) | 0.96 | 0.7 - 1.3 |  |
| 2 persons | 19/29 (65.5) | 0.92 | 0.6 - 1.3 |  |
| 3 persons | 12/21 (57.1) | 0.80 | 0.6 - 1.0 |  |
| 4 persons | 12/20 (60.0) | 0.84 | 0.6 - 1.2 |  |
| ≥ 5 persons | 20/23 (87.0) | 1.22 | 1.0 - 1.5 | **0.0101** |
| **Number of biological children** |  |  |  |  |
| None | 30/33 (90.9) | 1 |  |  |
| 1 child | 29/52 (55.8) | 0.61 | 0.5 -0.8 |  |
| 2 children | 20/30 (66.7) | 0.73 | 0.6 - 0.9 |  |
| 3 children | 8/13 (61.5) | 0.68 | 0.5 - 1.0 |  |
| ≥ 4 children | 9/12 (75.0) | 0.83 | 0.6 - 1.1 | **0.0002** |
| **Sexual behaviors** |  |  |  |  |
| **Age at first sex** |  |  |  |  |
| ≤ 15 | 33/50 (66.0) | 1 |  |  |
| 16 - 17 | 27/42 (64.3) | 0.97 | 0.8 - 1.2 |  |
| 18 - 19 | 23/33 (69.7) | 1.01 | 0.8 - 1.4 |  |
| ≥ 20 | 13/15 (86.7) | 1.31 | 1.0 - 1.6 | **0.0674** |
| **Sex work debut (age)** |  |  |  |  |
| ≤ 17 | 12/22 (54.6) | 1 |  |  |
| 18 - 21 | 33/47 (70.2) | 1.28 | 0.9 - 1.9 |  |
| 22 - 25 | 27/40 (67.5) | 1.24 | 0.9 - 1.7 |  |
| 26 - 29 | 12/17 (70.6) | 1.29 | 0.8 - 2.0 |  |
| ≥ 30 | 12/14 (85.7) | 1.57 | 1.1 - 2.3 | **0.1571** |
| **Involvement in sex work (years)** |  |  |  |  |
| ≤ 1 | 9/15 (60.0) | 1 |  |  |
| 2 | 15/21 (71.4) | 1.19 | 0.8 - 1.8 |  |
| 3 - 4 | 26/35 (74.3) | 1.24 | 0.8 - 2.8 |  |
| 5 - 9 | 32/44 (72.7) | 1.21 | 0.8 - 1.8 |  |
| ≥ 10 | 14/25 (56.0) | 0.93 | 0.6 - 1.5 | 0.6518 |
| **Number of clients (last day of work)** |  |  |  |  |
| ≤ 1 | 29/44 (65.9) | 1 |  |  |
| 2 to 3 | 33/52 (63.5) | 0.96 | 0.7 - 1.3 |  |
| 4 to 5 | 18/25 (72.0) | 1.05 | 0.7 - 1.5 |  |
| > 5 | 16/19 (84.2) | 1.28 | 1.0 - 1.6 | **0.1533** |
| **Number of clients (last seven days)** |  |  |  |  |
| ≤ 5 | 24/44 (54.6) | 1 |  |  |
| 6 - 10 | 18/21 (85.7) | 1.57 | 1.1 - 2.2 |  |
| 11 - 15 | 14/19 (73.7) | 1.35 | 1.0 - 1.8 |  |
| 16 - 20 | 17/25 (68.0) | 1.24 | 0.9 - 1.8 |  |
| ≥ 20 | 23/31 (74.2) | 1.36 | 1.0 - 1.8 | **0.0727** |
| **Money received for last sexual relation °** |  |  |  |  |
| ≤ 1500 | 20/24 (83.3) | 1 |  |  |
| 1501 - 2000 | 18/29 (62.1) | 0.75 | 0.6 - 1.0 |  |
| 2001 - 5000 | 35/56 (62.5) | 0.75 | 0.5 - 1.0 |  |
| > 5000 | 23/31 (74.2) | 0.96 | 0.7 - 1.2 | **0.1118** |
| **Prevention services** |  |  |  |  |
| **Use at least one SRH prevention services during sex work** | |  |  |  |
| No | 52/80 (65.0) | 1 |  |  |
| Yes | 44/60 (73.3) | 1.13 | 0.9 - 1.4 | 0.2320 |
| **Participate as peer worker in HIV and STI prevention activities** | |  |  |  |
| No | 88/127 (69.3) | 1 |  |  |
| Yes | 8/13 (61.4) | 0.89 | 0.6 - 1.4 | 0.5878 |
| **HIV screening at least once during lifetime** | |  |  |  |
| No | 2/4 (50.0) | 1 |  |  |
| Yes | 94/136 (69.2) | 1.38 | 0.7 - 2.9 | 0.3823 |
| **Contraception** |  |  |  |  |
| **Currently using hormonal contraception** | |  |  |  |
| No | 71/103 (68.9) | 1 |  |  |
| Yes | 25/37 (67.6) | 0.98 | 0.8 - 1.2 | 0.8676 |
| **Condom use (last seven days)** | |  |  |  |
| With clients | |  |  |  |
| Not always | 11/11 (100.0) | 1 |  |  |
| Always | 85/129 (65.9) | 0.66 | 0.6 – 0.8 | **<0.0001** |
| With non-paying partners | |  |  |  |
| Never/ Not always | 49/60 (81.7) | 1 |  |  |
| Always | 5/11 (45.5) | 0.56 | 0.3 - 0.9 |  |
| No sexual relation | 47/69 (60.9) | 0.75 | 0.6 - 0.9 | **0.0003** |

*Proportion of women with at least one pregnancy during the course of sex work

** p-value in the univariate analysis. P-values written in bold are <0.20

*** p-value in the multivariate analysis, adjusted for the year of the two different surveys; p-values written in bold are ≤0.05

**** *p-value, test for linear trend in the multivariate analysis,* adjusted for the year of the two different surveys; p-values written in bold are ≤0.05

*° In FCF*A (1 US dollars $\pm$= 500
